# Correlation between lenticulostriate arteries and white matter microstructure changes in patients with cerebral small vessel disease

**DOI:** 10.3389/fnins.2023.1202538

**Published:** 2023-09-25

**Authors:** Yukun Zhang, Peipei Chang, Na Liu, Yuhan Jiang, Ying Chu, Wei Du, Liangjie Lin, Bingbing Gao, Yuan Li, Mingrui Qu, Chao Yang, YanWei Miao

**Affiliations:** ^1^Department of Radiology, The First Affiliated Hospital, Dalian Medical University, Dalian, China; ^2^Department of Neurology, The First Affiliated Hospital, Dalian Medical University, Dalian, China; ^3^Philips Healthcare (China), Shanghai, China

**Keywords:** cerebral small vessel disease, lenticulostriate artery, vascular wall imaging, diffusion tensor imaging, tract-based spatial statistics

## Abstract

To explore the correlation between the number of lenticulostriate arteries (LSAs) and the white matter features in cerebral small vessel diseases (CSVD) by 3T magnetic resonance imaging (MRI). Seventy-one patients with diagnoses of CSVD were prospectively enrolled to undergo 3T MRI examination, including high-resolution vascular wall imaging (VWI) and diffusion tensor imaging (DTI). The LSAs were observed and counted on VWI, and the patients were divided into three groups according to the LSA counts. The presence of white matter hyperintensities (WMHs), lacunes, cerebral microbleeds (CMBs), and enlarged perivascular spaces (EPVS) was assessed in each patient, and a composite CSVD score was calculated. Periventricular and deep white matter hyperintensity (PVWMH, DWMH) volume ratios were obtained based on automatic segmentation. Fractional anisotropy (FA) and mean diffusivity (MD) were processed by using tract-based spatial statistics (TBSS) analysis. These parameters were compared among the three groups. Correlations between the LSA counts and white matter features were also analyzed. There were differences in WMHs (*P* = 0.001), CMBs (*P* < 0.001), EPVS (*P* = 0.017), composite CSVD scores (*P* < 0.001), PVWMH volume ratios (*P* = 0.001), DWMH volume ratios (*P* < 0.001), global FA (*P* = 0.001), and global MD (*P* = 0.002) among the three groups. There were correlations between the LSA counts and WMHs (*r* = −0.45, *P* < 0.001), CMBs (*r* = −0.44, *P* < 0.001), EPVS (*r* = −0.28, *P* = 0.020), the composite CSVD score (*r* = −0.52, *P* < 0.001), DWMH volume ratio (*r* = −0.47, *P* < 0.001), PWMH volume ratio (*r* = −0.34, *P* = 0.004), global FA (*r* = 0.36, *P* = 0.002), and global MD (*r* = −0.33, *P* = 0.005). Diabetes mellitus (OR 3.36, 95% CI 1.06–10.63; *P* = 0.039) and increased DWMH volume ratios (OR 1.04, 95% CI 1.00–1.08; *P* = 0.048) were independent risk factors for a decrease in LSA counts. TBSS analysis showed differences among the three groups in global FA and MD after adjusting for age and sex (*P* < 0.05). The LSA counts was associated with white matter microstructure changes in CSVD and has the potential to represent the extent of subcortical microvascular damage in CSVD patients.

## Introduction

Cerebral small vessel diseases (CSVD) are commonly observed by neuroimaging in elderly people and are now recognized as an important vascular contributor to dementia and cognitive decline, gait impairment, mood disturbance, and stroke (de Laat et al., 2010). The etiopathogenesis of CSVD involves arteriosclerosis and lipohyalinosis, with fibrinoid degeneration of perforating arteries, capillaries, and venules followed by compromise of the blood-brain barrier (De Silva and Faraci, 2020). Typical indicators of CSVD on magnetic resonance imaging (MRI) include white matter hyperintensities (WMHs), recent small subcortical infarcts (RSSIs), lacunes, enlarged perivascular spaces (EPVS), cerebral microbleeds (CMBs), and cortical superficial siderosis (cSS). However, these imaging changes mainly describe brain parenchyma lesions rather than the underlying small vessel changes.

Microstructural damage in the white matter is another important change in the pathophysiology of CSVD (Egle et al., 2021). Diffusion tensor imaging (DTI) is highly sensitive to white matter microstructural damage (Zeestraten et al., 2017). DTI parameters, such as fractional anisotropy (FA) and mean diffusivity (MD) are associated with microvascular damage (Zhuo et al., 2019), which are regarded as a sensitive MR imaging marker for monitoring the progression and assessing therapeutic interventions in patients with CSVD (Zeestraten et al., 2017). Tract-based spatial statistics (TBSS) can be applied to DTI analysis to negate the prominent contamination of data from cerebrospinal fluid (CBF) by focusing on the main fiber tracts (Metzler-Baddeley et al., 2012). TBSS-based image analysis has revealed widespread disruption of the white matter microstructure in CSVD patients who have developed depressive states (Gu et al., 2022) and apathy (Saleh et al., 2021).

Cerebral arterial small vessels are essential to maintain the optimum functioning of intricate white matter microstructure networks in CSVD (Wardlaw et al., 2013). The lenticulostriate arteries (LSAs) are the most common cerebral perforating arteries supplying blood to the basal ganglia and internal capsule regions, where 35 to 44% of ischemic and hemorrhagic strokes occur (Xie et al., 2021). The visualization of LSAs is crucial for understanding the mechanisms of microvascular pathologies (Zhang et al., 2019). Recently, high resolution vessel wall imaging (VWI) with 3.0T MRI has proven capable of non-invasively acquiring images of submillimeter-sized cerebral vessels (Zhang et al., 2019, Jiang et al., 2021; Xu et al., 2021), and patients with CSVD have shown decreased numbers of perforating arteries in the basal ganglia (Geurts et al., 2018; Chen et al., 2019). However, the concurrent relationships between white matter microstructure integrity and the LSAs in CSVD still have not been investigated.

In the present study, we hypothesized that the LSA counts have the potential to represent the extent of subcortical small vessel damage, and related to the damage of white matter in CSVD patients. Thus, this study aimed to use 3.0T MRI to quantitatively evaluate relationship between the LSA counts and multimodal MR markers of CSVD (the composite CSVD score, WMH volume, and DTI parameters) and to provide insight into the LSA counts as a marker in CSVD.

## Materials and methods

### Participants

**FIGURE 1:**
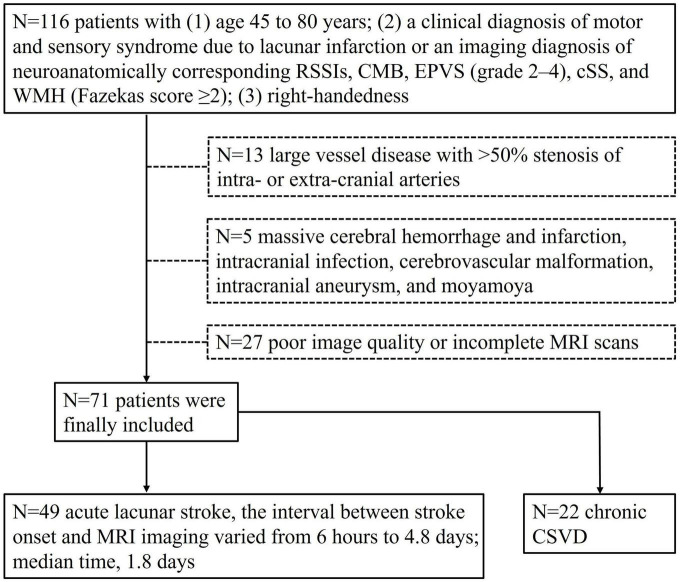
Inclusion and exclusion criteria flow chart of the study population. Seventy-one patients were enrolled.

This prospective study was approved by the ethics committee of the First Affiliated Hospital of Dalian Medical University (approval number: PJ-KS-KY-2022-302), and all participants gave written informed consent. A total of 116 patients with CSVD were enrolled in the study from January 2021 to July 2022. The patient diagnoses were confirmed by trained neurologists. The inclusion criteria were: (1) age 45 to 80 years; (2) a clinical diagnosis of motor and sensory syndrome due to lacunar infarction or an imaging diagnosis of neuroanatomically corresponding RSSIs, CMB, EPVS (grade 2–4), cSS, and WMH (Fazekas score ≥2) (Hassan et al., 2003); and (3) right-handedness (Gu et al., 2022). The exclusion criteria were: (1) large vessel disease with >50% stenosis of intra- or extra-cranial arteries; (2) severe cerebral disease, such as massive cerebral hemorrhage and infarction, intracranial infection, cerebrovascular malformation, intracranial aneurysm, moyamoya disease, etc.; (3) insufficient MRI image quality. The inclusion and exclusion criteria are showed in Figure 1.

### Clinical assessments

All patients underwent clinical assessments before MRI scanning. Demographics (age and gender) and cardiovascular risk factors including history of hypertension, diabetes mellitus, hyperlipidemia, coronary artery disease, cerebrovascular disease, and smoking were recorded. Current smokers or those who stopped smoking within 1 year before the index stroke were all considered as smokers. Laboratory findings were recorded, including total cholesterol, triglyceride, high-density lipoprotein (HDL) cholesterol, low-density lipoprotein (LDL) cholesterol, uric acid, and glucose. Baseline neurological deficits were assessed using the National Institutes of Health Stroke Scale (NIHSS).

### Image acquisition protocol

Magnetic resonance imaging data for all subjects were acquired using a 3.0T MRI scanner (Ingenia CX, Philips Healthcare, Best, Netherlands) equipped with a 32-channel head coil. The scanning protocols included T2-weighted imaging (T2WI, TR/TE = 2345/122 ms; field of view (FOV) = 230 mm × 230 mm; voxel size = 0.60 mm × 0.60 mm × 6.50 mm; and scan time of 56 s), T2 fluid-attenuated inversion recovery (T2 FLAIR, TR/TE = 9000/126 ms; FOV = 230 mm × 187 mm; voxel size = 0.75 mm × 1.04 mm × 6.50 mm; and scan time of 81 s), T1-weighted imaging (T1WI, TR/TE = 2162/15 ms; FOV = 230 mm × 217 mm; voxel size = 0.80 mm × 0.89 mm × 6.50 mm; and scan time of 45 s), diffusion weighted imaging (DWI, TR/TE of 2217/58 ms; FOV of 184 mm × 226 mm; voxel size = 1.60 mm × 2.04 mm × 5.00 mm; two *b*-values of 0 and 1000 s/mm^2^; and scan time of 20 s), susceptibility weighted imaging (SWI, TR/TE = 29/5.6 ms; FOV = 230 mm × 187 mm; voxel size = 0.60 mm × 0.90 mm × 2.50 mm; and scan time of 102 s), three-dimensional time-of-flight magnetic resonance angiography (3D TOF MRA, TR/TE = 23/3.5; FOV = 200 mm × 180 mm; voxel size = 0.45 mm × 0.68 mm × 1.20 mm; scan time of 146 s), VWI (TR/TE = 900/30 ms; FOV = 220 mm × 181 mm; voxel size = 0.50 mm × 0.50 mm × 0.50 mm; and scan time of 480 s), and DTI (TR/TE = 6000/92 ms; FOV = 256 mm × 256 mm; voxel size = 2.00 mm × 2.00 mm × 2.00 mm; and scan time of 403 s). Each DTI dataset included 64 non-collinear spatial directions at *b*-value = 1,000 s/mm^2^, and one baseline image at b = 0 s/mm^2^.

### Analysis of lenticulostriate arteries

**FIGURE 2:**
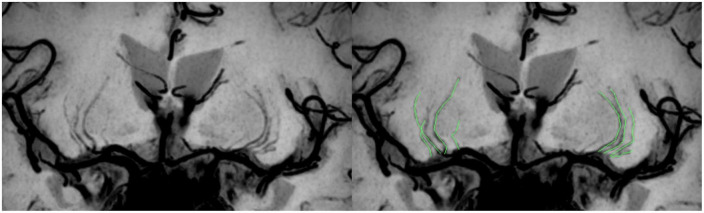
Schematic diagram of the quantitative analysis of the LSAs. High-resolution VWI is reconstructed by coronal MPR and MinIP, allowing clear visualization of the LSAs. There are 4 LSAs visible on each side in the patient shown here; the LSAs delineated in green in the figure on the right.

Vascular wall imaging was restructured on the Philips workstation (Intellispace portal version 9.0, ISP v9.0) into a coronal plane by multiplanar reformation (MPR), followed by a minimum intensity projection (MinIP) to maximize the visibility of the LSAs. Only LSAs longer than 5 mm on the M1 segment of the middle cerebral artery (MCA) were counted. Each LSA branch was counted individually when the branch emitted within 5 mm of the MCA origin on the LSA trunk, but only the longest branches were counted when the branch was with more distal, based on the finding that >70% of the branches originate from the common LSA branch (Xie et al., 2021; Figure 2). Two radiologists, one with 10 years (observer 1) and the other with 5 years (observer 2) of experience in neuroradiology, independently performed quantification to evaluate interobserver agreement. Observer 1 reevaluated the measurements after a period of 2 months to assess intraobserver agreement.

The LSAs counts of both sides of the CSVD cohort were tallied, revealing a maximum count of 10 and a minimum count of 2. Consequently, based on the bilateral LSA counts, the CSVD cohort was evenly categorized into three groups in descending order (Group 1: 10 to 8 LSAs; Group 2: 7 to 5 LSAs; Group 3: 4 to 2 LSAs) (Chen et al., 2016).

### Assessment of cerebral small vessel disease

Recent small subcortical infarcts were defined as ovoid or rounded lesions and were small (>3 and <20 mm) in diameter, and were carefully distinguished from WMHs. Lacunes were assessed with T2 FLAIR and were small (>3 and <20 mm in diameter) ovoid or round lesions containing CBF signal intensity, and there was no signal enhancement on DWI. CMBs were defined as small (<5 mm in diameter), homogeneous, round foci of low signal intensity on SWI (Staals et al., 2014). RSSIs, lacunes, and CMBs lesions in the cerebellum, brainstem, basal ganglia, white matter, or cortico-subcortical junction were recorded. Deep (D) and periventricular (PV) WMHs were both assessed according to the Fazekas scale from 0 to 3, using T2 FLAIR imaging (Fazekas et al., 1987). EPVS was defined as small (<3 mm) punctate and linear hyperintensity on T2WI in the basal ganglia or centrum semiovale, and they were scored on a validated semiquantitative scale from 0 to 4. We only counted EPVS in the basal ganglia as specifically associated with CSVD (Doubal et al., 2010). The aforementioned two radiologists independently assessed these lesions in all patients.

A composite CSVD score (Staals et al., 2014) on an ordinal scale from 0 to 4 was determined by the presence of RSSIs or lacunes (1 point), presence of CMBs (1 point), the presence of moderate to severe (grade 2 to 4) EPVS (1 point), and the presence of DWMHs (with Fazekas score 2 or 3) or PVWMHs extending into the deep white matter (Fazekas score 3) (1 point).

### MRI data pre-processing analysis

#### White matter hyperintensity volume ratios

The PVWMH and DWMH volume ratios, determined as the ratio of the WMH volumes to the normalized brain volume, were obtained by using a deep learning-based automated WMH segmentation procedure in the uAI Research Portal V1.1 (Shanghai United Imaging Intelligence, Co., Ltd.) (Zhu et al., 2022). This procedure consisted of three steps. 1. Data pre-processing: T1WI and T2WI images were registered to the FLAIR images via rigid and affine transformations computed to determine the territory of WMH and differentiate WMH from other cerebral lesions, such as lacunes, EPVS, and large infarctions; 2. Image segmentation: 2D VB-Net is used as the backbone of the network to handle the WMH segmentation problem; 3. Quantification: The number of voxels occupied by WM lesions was calculated and multiplied by the voxel resolution of FLAIR images to obtain the WMH volume (mm^3^), and the WMH was subclassified into PWMH and DWMH.

#### Diffusion tensor imaging

The TBSS pipeline in FSL 5.0.9 was used to perform a whole-brain analysis for all participants (Lenka et al., 2020). First, the data were corrected for motion and eddy currents by using Eddy, and the *dtifit* tool was used to fit the diffusion tensor at each voxel (single tensor model) to produce FA and MD maps. Second, FA maps for all participants were non-linearly aligned to the FMRIB-58 FA map from the Montreal Neurological Institute (MNI) template space. Third, following deformable registration, the mean FA skeleton (skeleton threshold = 0.2) was computed, which represented the center of the white matter tracts common to all CSVD patients. In addition, the deformation fields from the FA maps were used for registration of the MD maps, and the registered maps were projected onto the mean FA skeleton.

Statistical significance was assessed using the FSL permutation test. The general linear model (GLM) was performed as the statistical modeling of voxel-wise statistical analysis, and pairwise and three-way comparisons among the three groups were performed with the GLM; the covariates included sex and age. The significance level of the two DTI parameters (FA, MD) was *P* < 0.05 [5000 permutations, strong control of family wise error (FWE) correction for multiple comparisons correction using the threshold-free cluster enhancement]. Finally, we extracted the average whole-brain FA and MD and displayed the differences between groups by using the John Hopkins University (JHU) white-matter tractography atlas.

#### Statistical analysis

All statistical analyses were performed with statistical package for social science (SPSS) version 26.0. Intra- and interrater agreement of the visual assessments and measurements were checked by using intraclass correlation coefficients (ICCs) or Kappa test. The normality of the data was analyzed by using the Kolmogorov-Smirnov test. Normally distributed data were expressed as means ± standard deviation (SD) and non-normally distributed continuous variables were expressed as median (interquartile ranges, IQR) and were compared using the Wilcoxon rank-sum test. Qualitative data were summarized as count (percentage) and were compared using the χ^2^ test. A *post hoc* analysis was performed for comparisons between groups, and the Bonferroni’s correction was used for multiple comparisons. One-way ANOVA or Kruskal-Wallis test as appropriate was used among the three groups. Pearson and Spearman correlation was performed to check for relationships between the LSA counts and multimodal MR parameters of CSVD. Multivariable Logistic regression was tested to find the independent clinical factors influencing the LSA counts, and was adjusted by gender, hypertension, diabetes mellitus, hyperlipidemia, smoking, cerebrovascular disease, coronary artery disease, and NIHSS. All statistical tests were two-sided with *P* < 0.05 considered significant.

## Results

### Patient characteristics

Total of 71 patients with CSVD (44 males, 27 females; mean age 64.90 ± 9.40 years old) were included in the study. For the LSA counts, the intra and interobserver ICCs were 0.985 and 0.962 (all *P* < 0.05). For the intraobserver agreement in assessment of CSVD, the Kappa was 0.801, 0.660, 0.831, and 0.683 for WMHs, lacunes, CMBs, and EPVS, respectively (all *P* < 0.05). For the interobserver agreement in assessment of CSVD, the Kappa was 0.688, 0.654, 0.634, and 0.590 for WMHs, lacunes, CMBs, and EPVS, respectively (all *P* < 0.05). There was no significant differences in age, sex, vascular risk factors, and laboratory findings among the three groups (*P* > 0.05, Table 1).

**TABLE 1:** Baseline characteristics of the three groups.

	Group 1 (*n* = 19)	Group 2 (*n* = 30)	Group 3 (*n* = 22)	*P*-value
**Classification of CSVD**				
Acute lacunar stroke, *n* (%)	13 (68.4%)	18 (60%)	18 (81.8%)	0.243
**Demographics**				
Man, *n* (%)	10 (52.6%)	19 (63.3%)	15 (68.2%)	0.585
Age, mean ± SD	62.05 ± 11.58	65.20 ± 8.52	66.95 ± 7.71	0.427
**Risk factor, *n* (%)**				
Hypertension	12 (63.2%)	27 (90.0%)	17 (77.3%)	0.082
Diabetes mellitus	4 (21.1%)	10 (33.3%)	12 (54.6%)	0.078
Hyperlipidemia	3 (15.8%)	4 (13.3%)	3 (13.6%)	0.969
Smoking	9 (47.4%)	11 (36.7%)	10 (45.5%)	0.715
History of cerebrovascular diseases	0	7 (23.3%)	2 (9.1%)	0.050
Coronary artery disease	1 (5.3%)	6 (20.0%)	4 (18.2%)	0.354
NIHSS, mean ± SD	2.42 ± 2.06	3.13 ± 2.36	3.18 ± 3.33	0.372
**Laboratory finding, mean ± SD**				
Total cholesterol, mmol/L	4.57 ± 0.80	4.56 ± 0.95	4.75 ± 1.20	0.792
Triglyceride, mmol/L	1.67 ± 1.07	1.35 ± 0.49	1.55 ± 0.66	0.725
HDL cholesterol, mmol/L	1.23 ± 0.26	1.15 ± 0.22	1.14 ± 0.32	0.342
LDL cholesterol, mmol/L	2.54 ± 0.58	2.53 ± 0.67	2.63 ± 0.86	0.888
Uric acid, μmol/L	316.21 ± 81.86	343.47 ± 80.82	363.68 ± 86.54	0.209
Glucose, mmol/L	5.56 ± 0.84	6.38 ± 2.66	6.29 ± 2.37	0.924

NIHSS, national institutes of health stroke scale; HDL cholesterol, high-density lipoprotein cholesterol; LDL cholesterol, low-density lipoprotein cholesterol.

### Comparison of imaging features among the three groups

**FIGURE 3:**
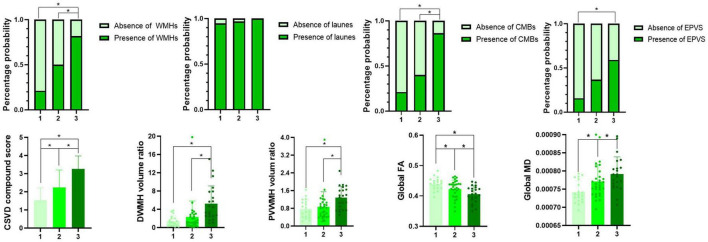
Multimodal magnetic resonance imaging markers among groups 1–3. *Indicates a significant difference vs. two other groups (*P* < 0.05).

Comparisons of the multimodal MR markers of CSVD among the three groups are shown in Figure 3 and Table 2. There were significant differences in WMHs (*P* = 0.001), CMBs (*P* < 0.001), EPVS (*P* = 0.017), composite CSVD score (*P* < 0.001), PVWMH volume ratio (*P* = 0.001), DWMH volume ratio (*P* < 0.001), global FA (*P* = 0.001), global MD (*P* = 0.002), and LSA counts (*P* < 0.001) among the three groups. Correlation analysis was performed between MR parameters of CSVD and the LSA counts, and the LSA counts was significantly correlated with WMHs (*r* = −0.45, *P* < 0.001), CMBs (*r* = −0.44, *P* < 0.001), EPVS (*r* = −0.28, *P* = 0.020), the composite CSVD score (*r* = −0.52, *P* < 0.001), DWMH volume ratio (*r* = −0.47, *P* < 0.001), PWMH volume ratio (*r* = −0.34, *P* = 0.004), global FA (*r* = 0.36, *P* = 0.002), and overall MD (*r* = −0.33, *P* = 0.005). In a logistic regression model, the increase of DWMH volume ratios (odds ratio [OR] 1.04, 95% confidence interval [CI] 1.00–1.08; *P* = 0.048) and occurrence of diabetes mellitus (OR 3.36, 95% CI 1.06–10.63; *P* = 0.039) were independently associated with the decrease of the LSA counts (Table 3).

**TABLE 2:** Multimodal magnetic resonance neuroimaging findings among the three groups.

	Group 1 (*n* = 19)	Group 2 (*n* = 30)	Group 3 (*n* = 22)	*P*-value
Presence of WMHs, *n* (%)	4 (21.1%)	15 (50.0%)	18 (81.8%)	**0**.**001**
Presence of lacunes, *n* (%)	18 (94.7%)	29 (96.7%)	22 (100.0%)	0.582
Presence of CMBs, *n* (%)	4 (21.1%)	12 (40.0%)	19 (86.4%)	<**0**.**001**
Presence of EPVS, *n* (%)	3 (15.7%)	11 (36.7%)	13 (59.1%)	**0**.**017**
Composite CSVD score, median (IQR)	1 (1–2)	2 (1.25–3)	3 (3–4)	<**0**.**001**
PVWMH volume (%), median (IQR)	0.65 (0.43–1.07)	0.82 (0.44–0.93)	1.34 (0.78–1.61)	**0**.**001**
DWMH volume (%), median (IQR)	1.16 (0.48–2.00)	1.26 (0.79–2.63)	3.95 (2.54–7.19)	<**0**.**001**
Global FA, median (IQR)	0.44 (0.43–0.45)	0.43 (0.40–0.44)	0.41 (0.39–0.43)	**0**.**001**
Global MD (× 10^–3^ mm^2^/s), mean ± SD	0.74 ± 0.03	0.77 ± 0.05	0.79 ± 0.05	**0**.**002**
Number of LSAs, median (IQR)	8 (8–9)	6 (5– 6.75)	4 (4–4)	<**0**.**001**

WMHs, white matter hyperintensities; CMBs, cerebral microbleeds; EPVS, enlarged perivascular spaces; PVWMH, periventricular white matter hyperintensity; DWMH, deep white matter hyperintensity; FA, fractional anisotropy; MD, mean diffusivity; LSAs, lenticulostriate arteries; IQR, interquartile ranges. The bold values indicate significant statistical differences (*P* < 0.05).

**TABLE 3:** Univariate and multivariable logistic regression analysis of relative factors for three groups.

	Univariate		Multivariable	
	OR (95% CI)	*P*-value	OR (95% CI)	*P*-value
Man	1.59 (0.65–3.87)	0.312	2.49 (0.53–11.69)	0.247
Age	1.04 (0.99–1.09)	0.086	0.99 (0.99–1.01)	0.579
Hypertension	1.82 (0.63–5.28)	0.272	0.77 (0.19–3.11)	0.714
Diabetes mellitus	2.91 (1.14–7.39)	**0**.**025**	3.36 (1.06–10.63)	**0**.**039**
Hyperlipidemia	0.89 (0.26–3.06)	0.850	0.64 (0.11–3.56)	0.607
Smoking	0.97 (0.41–2.32)	0.944	0.79 (0.19–3.34)	0.750
History of cerebrovascular disease	1.48 (0.40–5.45)	0.555	0.83 (0.13–5.27)	0.845
Coronary artery disease	1.84 (0.55–6.17)	0.320	5.04 (0.89–28.50)	0.067
NIHSS	1.01 (0.99–1.03)	0.388	1.00 (0.98–1.03)	0.954
Presence of WMHs	6.81 (2.52–18.40)	<**0**.**001**	2.47 (0.30–20.33)	0.400
Presence of CMBs	8.66 (3.09–24.30)	<**0**.**001**	6.04 (0.65–56.12)	0.114
Presence of EPVS	3.99 (1.51–10.10)	**0**.**005**	1.37 (0.15–12.47)	0.781
Composite CSVD score	1.13 (1.08–1.19)	<**0**.**001**	1.04 (0.86–1.24)	0.718
PVWMH volume (%)	1.12 (1.02–1.22)	**0**.**016**	0.90 (0.75–1.09)	0.283
DWMH volume (%)	1.03 (1.01–1.06)	**0**.**002**	1.04 (1.00–1.08)	**0**.**048**
Global FA	0.73 (0.61–0.87)	<**0**.**001**	0.79 (0.44–1.40)	0.413
Global MD	6.12 (2.02–18.49)	**0**.**001**	0.17 (0.01–5.96)	0.330

OR, odds ratio; CI, confidence interval; WMHs, white matter hyperintensities; CMBs, cerebral microbleeds; EPVS, enlarged perivascular spaces; PVWMH, periventricular white matter hyperintensity; DWMH, deep white matter hyperintensity; FA, fractional anisotropy; MD, mean diffusivity; LSAs, lenticulostriate arteries. The bold values indicate significant statistical differences (*P* < 0.05).

### Diffusion tensor imaging whole-brain analysis

**FIGURE 4:**
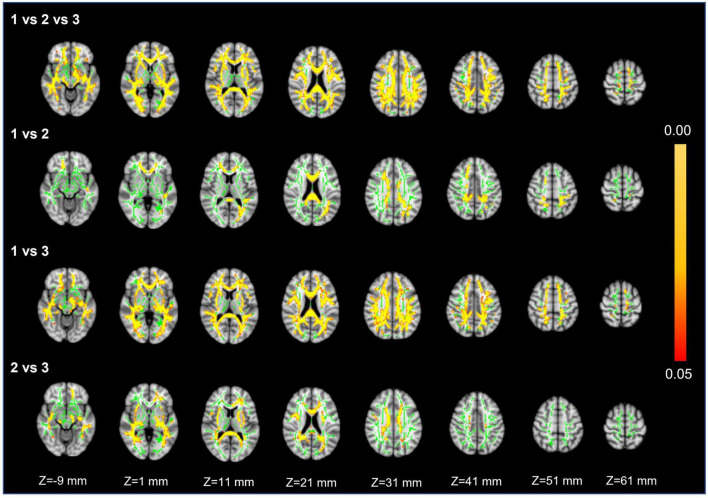
Fractional anisotropy. First row: results of ANCOVA analysis of the FA parameters across three groups. Second to fourth rows: results of two-sample *t*-test of the FA parameters between Groups 1 vs. 2, 1 vs. 3, and 2 vs. 3. The green color represents the mean FA skeleton of all participants. The red-yellow colormap represents the *P*-values in regions with statistical significance [*P* < 0.05, corrected by threshold free cluster enhancement (TFCE)].

**FIGURE 5:**
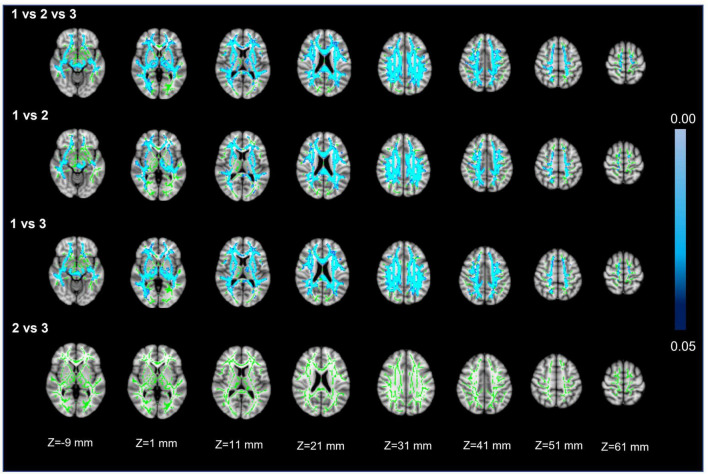
Mean diffusivity. First row: results of ANCOVA analysis of the MD parameters across three groups. Second to fourth rows: results of two-sample *t*-test of the MD parameters between Groups 1 vs. 2, 1 vs. 3, and 2 vs. 3. The green color represents the mean MD skeleton of all participants. The blue colormap represents the P-values in regions with statistical significance [*P* < 0.05, corrected by threshold free cluster enhancement (TFCE)].

Voxel white matter integrity differences were evaluated by using the TBSS method to analyze DTI parameters (Figures 4, 5). Comparisons among the three groups showed significant differences in both FA and MD in a wide range of WM tracts. Meanwhile, compared with Group 1, Groups 2 and 3 showed significantly decreased FA and increased MD in a wide range of WM tracts. Compared with Group 2, Group 3 showed significantly decreased FA in multiple WM tracts but no significant difference in MD. The extensive WM tracts described above included anterior thalamic radiation (ATR), corticospinal tract (CST), cingulum [cingulate gyrus (CCG)], cingulum (hippocampus), forceps major (FMA), forceps minor (FMI), inferior fronto-occipital fasciculus (IFOF), inferior longitudinal fasciculus (ILF), superior longitudinal fasciculus (SLF), superior longitudinal fasciculus (temporal part [TP]), and uncinate fasciculus (UF) (Supplementary Tables 1, 2).

## Discussion

This study examined the correlation between the number of LSAs and multimodal MR imaging parameters of CSVD. Three groups stratified by decreasing LSA counts showed significant differences in WMHs, CMBs, EPVS, composite CSVD scores, PVWMH and DVWMH volume ratios, and global FA and MD values. Correlation analysis showed that the LSA counts was negatively correlated with WMHs, CMBs, EPVS, the composite CSVD score, the DWMH and PWMH volume ratios, and global MD, while positively correlated with global FA. The increase of DWMH volume ratio and occurrence of diabetes mellitus were independently associated with decrease of the LSA counts. In addition, the comparison of FA and MD based on TBSS analysis among the three groups showed different degrees of damage to fiber tracts in almost all parts of the brain.

The finding of negative correlations of the LSA counts with WMHs, CMBs, EPVS, and the composite CSVD score obtained from 3.0T MR imaging are similar to the result of a previous DSA-based study (Chen et al., 2019). By using an automated procedure (Joo et al., 2022), we found that the PVWMH and DWMH volume ratios in CSVD patients gradually increased as the number of LSAs decreased, and DWMH is the independent influencing factor of the LSA counts. PVWMHs are mainly caused by reduced perfusion in the watershed region, while DWMH progression has mainly been attributed to occlusion of deep penetrating vessels (Promjunyakul et al., 2018), which reflects the relationship between the LSAs and other deep penetrating arteries. Furthermore, occurrence of diabetes mellitus is also the independent clinical factor influencing the LSA counts, which is similar to the results of a previous study (Yashiro et al., 2018). This can be explained by mechanisms underlying hyperglycemia induced microvascular complications, mainly resulting from damage to endothelial cells caused by oxidative stress.

Cerebral small vessel diseases is a dynamic and whole-brain disorder, and quantitative MRI methods, such as DTI, T1 mapping, and dynamic contrast-enhanced MRI, can increasingly reveal abnormal tissue in perilesional zones of normal-appearing white matter around WMHs and lacunes (Munoz Maniega et al., 2017, van Leijsen et al., 2018). The present study found that as the number of LSAs decreased in CSVD patients, the whole-brain FA decreased and the whole-brain MD increased. MD is an orientation-independent marker of white matter ultrastructure that can describe the overall extent of water diffusion, while FA provides information on the directionality of the diffusion tensor and therefore shows the organization and damage of the ultrastructure. As CSVD progresses, these results probably reflect the loss of cerebrovascular autoregulation and cerebrovascular reactivity functions, which leads to diffuse endothelial failure, and thus, worsening arteriosclerosis, lipohyalinosis, and fibrinoid degeneration (Wardlaw et al., 2019). These further lead to thickened vessel walls and lumen occlusion of small vessels in the cerebral cortex, deep medulla, and basal ganglia regions, which could contribute to reduced perfusion and concomitant demyelination, axon loss, and gliosis (Sam et al., 2016, Promjunyakul et al., 2018). These cerebrovascular degenerations will manifest as decreased FA and increased MD on MR imaging (Bagi et al., 2022), and may eventually progress to the corresponding DWMH.

For FA and MD based on TBSS analysis, the comparison between Groups 1 and 3 showed a wider range of damage to fiber tracts vs. the comparisons between Groups 2 and 3 or Groups 1 and 2 (Figures 4, 5). This suggests that the loss of visible LSAs can be correlated with more serious damage to the white matter ultrastructure. The range of different regions was smaller for FA and larger for MD in Groups 1 vs. 2, but the result for Groups 2 vs. 3 was the opposite (Figures 4, 5). It is reported that MD may be more sensitive for detecting mild damage, while FA captures more severe damage (Cubon et al., 2011). As such, a smaller decrease in the LSA counts (Groups 1 vs. 2) may reflect mild damage to the white matter ultrastructure while a larger decrease (Groups 2 vs. 3) reflects more severe damage. Furthermore, we found that the differential cluster of all comparisons for MD and FA involved almost all of the WM tracts (Supplementary Tables 1, 2). CST passing through the LSA blood supply area was directly affected by LSAs (Konishi et al., 2005), while other WM tracts were not directly related to LSA counts, which further suggests that a change in the LSA counts represents a change in the functional status of the whole-brain perforator artery and arteriole. Therefore, the LSAs have the potential to serve as an imaging marker to capture early microvascular pathological changes in CSVD before permanent parenchymal damage occurs (van den Brink et al., 2022).

Several issues limited the interpretation of the current results. Firstly, the sample size could be expanded for further analysis. Secondly, there were subjective approaches when measuring the number of the LSAs. Advanced automated segmentation and reconstruction of LSAs should be introduced to improve measurement accuracy. Thirdly, it is difficult to ascertain the causation between LSAs and multimodal MR parameters in this cross-sectional study, and a longitudinal study will be performed in the future to further evaluate the potential of LSA counts as a CSVD marker. Lastly, none of the CSVD patients completed a detailed neuropsychological assessment, and thus the relationship between the LSA counts and cognitive functions could not be clarified.

## Conclusion

In conclusion, on 3T MR imaging in patients with CSVD, lower LSA counts were associated with occurrence of diabetes mellitus, larger DWMH volume ratios, and a higher degree of fiber tract damage in most brain regions, as manifested by lower FA values and higher MD values. This study shows that the LSA counts has the potential to represent the extent of subcortical microvascular damage of brain tissue in CSVD patients.

## Data availability statement

The original contributions presented in this study are included in the article/Supplementary material, further inquiries can be directed to the corresponding authors.

## Ethics statement

The studies involving humans were approved by the Ethics Committee of The First Affiliated Hospital of Dalian Medical University. The studies were conducted in accordance with the local legislation and institutional requirements. The participants provided their written informed consent to participate in this study. Written informed consent was obtained from the individual(s) for the publication of any potentially identifiable images or data included in this article.

## Author contributions

YZ: Conceptualization, Methodology, Formal analysis, Writing – original draft. PC: Writing – review and editing, Formal analysis, Validation. NL: Writing – review and editing, Formal analysis, Validation. YJ: Software, Methodology, Writing – review and editing. YC: Resources, Data curation. WD: Software, Writing – review and editing. LL: Writing – review and editing, Formal analysis. BG: Software, Methodology. YL: Data curation. MQ: Data curation. CY: Writing – Review and editing, Supervision. YM: Funding acquisition, Writing – review and editing.
