# Supplementary material for: Correlation between lenticulostriate arteries and white matter microstructure changes in patients with cerebral small vessel disease
Source: Front Neurosci. 2023 Sep 25;17:1202538. doi: 10.3389/fnins.2023.1202538 (PMC10560852; doi:10.3389/fnins.2023.1202538)
Supplement: Supplementary file 1 [file Data_Sheet_1.DOCX]

Supplementary Table 1. Cluster sizes and locations for voxels with significantly decreased FA among three groups and in groups 2 vs. 1, 3 vs. 1, and 3 vs. 2. Any cluster with low voxels (< 950) has been excluded.

| Group | Cluster index | Atlas region  (Percentage probability of the cluster) | Cluster size (voxels) | JHU-WM Tractography Atlas | | | *P* -value |
| --- | --- | --- | --- | --- | --- | --- | --- |
|  |  |  |  | **X** | **Y** | **X** |  |
| 1 vs. 2 vs. 3 | 1 | ATR_L:0.871977; ATR_R:0.693603  CST_L:0.799098; CST_R:0.394608  Cingulum (CCG)_L:0.404171  Cingulum (CCG)_R:0.214241  Cingulum (hippocampus)_L:0.00806012  Cingulum (hippocampus)_R:0.0703617  FMA:1.30969; FMI:3.55256  IFOF_L:1.82548; IFOF_R:2.42576  ILFL:1.51275; ILFR:1.26938  SLF_L:1.96654; SLF_R:1.42863  UF_L:0.471527; UF_R:0.306097  SLF-TP_L:0.931386; SLF-TP_R:0.556975 | 53225 | 95 | 147 | 88 | <0.001 |
| 1 vs. 2 | 1 | ATR_L:0.183414; ATR_R:0.945954  CST_L:0.389657; CST_R:0.248175  Cingulum (CCG)_L:0.43982  Cingulum (CCG)_R:0.13791  Cingulum (hippocampus)_L:0.000465911  Cingulum (hippocampus)_R:0.0135114  FMA:0.405964; FMI:5.61252  IFOF_L:0.00465911; IFOF_R:0.489828  ILFL:0.0264016; ILFR:0.00279547  SLF_L:0.264637; SLF_R:0.161516  UF_L:0.00279547; UF_R:0.505047  SLF-TP_L:0.004659; SLF-TP_R:0.016306 | 6439 | 94 | 126 | 97 | 0.029 |
|  | 2 | ATR_L:0.275676; ATR_R:0.00623701  Cingulum (hippocampus)_L:0.00748441  FMA:4.87651; FMI:0.00997921  IFOF_L:5.26154; ILFL:9.21414  SLF_L:5.74179; UF_L:0.167983  SLF-TP_L:2.89439 | 2405 | 124 | 66 | 76 | 0.043 |
| 1 vs. 3 | 1 | ATR_L:0.908613; ATR_R:0.700751  CST_L:0.803224; CST_R:0.389137  Cingulum (CCG)_L:0.424174  Cingulum (CCG)_R:0.219088  Cingulum (hippocampus)_L:0.00749071  Cingulum (hippocampus)_R:0.0716711  FMA:1.36021; FMI:3.73751  IFOF_L:1.92006; IFOF_R:2.48208  ILFL:1.5476; ILFR:1.26587  SLF_L:2.03709; SLF_R:1.44603  UF_L:0.487336; UF_R:0.267329  SLF-TP_L:0.964664; SLF-TP_R:0.551236 | 50062 | 97 | 99 | 36 | <0.001 |
| 2 vs. 3 | 1 | ATR_L:1.0969; ATR_R:0.10414  CST_L:1.53787; CST_R:0.836644  Cingulum (CCG)_L:0.0529051  Cingulum (CCG)_R:0.0704474  Cingulum (hippocampus)_L:0.00891034  Cingulum (hippocampus)_R:0.122146  FMA:3.01596; FMI:0.00501207  IFOF_L:3.46334; IFOF_R:4.86263  ILFL:2.8153; ILFR:2.82467  SLF_L:1.02636; SLF_R:1.92797  UF_L:0.136161; UF_R:0.179413  SLF-TP_L:0.737795; SLF-TP_R:1.01392 | 10774 | 112 | 108 | 69 | 0.016 |
|  | 2 | ATR_L:2.36274; ATR_R:0.209351  CST_L:0.03731; CST_R:0.00829111  Cingulum (CCG)_L:0.833947  Cingulum (CCG)_R:0.508521  Cingulum (hippocampus)_R:0.00829111  FMI:12.2748; IFOF_L:2.78996  IFOF_R:0.00437586; SLF_L:0.0292492  SLF_R:0.0428374; UF_L:1.83924  SLF-TP_L:0.069092; SLF-TP_R:0.018655 | 4342 | 94 | 111 | 97 | 0.029 |

Specific regions are derived from the JHU white matter atlas; TBSS: tract-based spatial statistics; FA: fractional anisotropy; ATR: anterior thalamic radiation; CST: corticospinal tract; CCG: cingulate gyrus; CH: cingulate hippocampus; FMA: forceps major; FMI: forceps minor; IFOF: inferior fronto-occipital fasciculus; ILF: inferior longitudinal fasciculus; SLF: superior longitudinal fasciculus; UF: uncinate fasciculus; SLF-TP: superior longitudinal fasciculus (temporal part).

Supplementary Table 2. Cluster sizes and locations for voxels with significantly increased MD among three groups and in groups 2 vs. 1 and 3 vs. 1. Any cluster with low voxels (< 950) has been excluded.

| Group | Cluster index | Atlas region  (Percentage probability of the cluster) | Cluster size (voxels) | JHU-WM Tractography Atlas | | | *P* -value |
| --- | --- | --- | --- | --- | --- | --- | --- |
|  |  |  |  | **X** | **Y** | **X** |  |
| 1 vs. 2 vs. 3 | 1 | ATR_L:1.4514; ATR_R:1.07454;  CST_L:1.21355; CST_R:0.975963;  Cingulum (CCG)_L:0.502058;  Cingulum (CCG)_R:0.177148;  Cingulum(hippocampus)_L:0.00638209; Cingulum(hippocampus)_R:0.0377955; FMA:0.639283; FMI:2.86045;  IFOF_L:1.5319; IFOF_R:2.45927;  ILFL:0.8855; ILFR:1.06287;  SLF_L:2.29875; SLF_R:2.12973;  UF_L:0.501223; UF_R:0.328051;  SLF-TP_L:0.949281; SLF-TP_R:0.788635 | 50297 | 47 | 114 | 51 | 0.001 |
| 1 vs. 2 | 1 | ATR_L:1.23204; ATR_R:0.859365  CST_L:1.15611; CST_R:0.556483  Cingulum (CCG)_L:0.582429  Cingulum (CCG)_R:0.217937  Cingulum(hippocampus)_L:0.00645977  Cingulum (hippocampus)_R:0.0362824  FMA:0.481603; FMI:2.9975  IFOF_L:1.13999; IFOF_R:2.58994  ILFL:0.608484; ILFR:1.02527  SLF_L:2.77668; SLF_R:2.45819  UF_L:0.352138; UF_R:0.394181  SLF-TP_L:1.09205; SLF-TP_R:0.894921 | 37153 | 121 | 88 | 83 | 0.001 |
| 1 vs. 3 | 1 | ATR_L:1.42459; ATR_R:1.0974  CST_L:1.18589; CST_R:0.974498  Cingulum (CCG)_L:0.332271  Cingulum (CCG)_R:0.183636  Cingulum (hippocampus)_L:0.00575036  Cingulum (hippocampus)_R:0.0393566  FMA:0.644728; FMI:2.96683  IFOF_L:1.59458; IFOF_R:2.56237  ILFL:0.912099; ILFR:1.09428  SLF_L:2.39023; SLF_R:2.21639  UF_L:0.522762; UF_R:0.342271  SLF-TP_L:0.990437; SLF-TP_R:0.823697 | 47997 | 128 | 120 | 46 | <0.001 |

Specific regions are derived from the JHU white matter atlas; TBSS: tract-based spatial statistics; MD, mean diffusivity. ATR: anterior thalamic radiation; CST: corticospinal tract; CCG: cingulate gyrus; CH: cingulate hippocampus; FMA: forceps major; FMI: forceps minor; IFOF: inferior fronto-occipital fasciculus; ILF: inferior longitudinal fasciculus; SLF: superior longitudinal fasciculus; UF: uncinate fasciculus; SLF-TP: superior longitudinal fasciculus (temporal part).
